# Initial Experience of Self-Expanding Metal Ureteral Stent in Recurrent Ureteral Stricture After Ureteroplasty

**DOI:** 10.3389/fsurg.2021.765810

**Published:** 2021-12-21

**Authors:** Xiaoshuai Gao, Jixiang Chen, Zhongyu Jian, Menghua Wang, Wei Wang, Liao Peng, Zhenghuan Liu, Xin Wei

**Affiliations:** Department of Urology, Institute of Urology (Laboratory of Reconstructive Urology), West China Hospital, Sichuan University, Chengdu, China

**Keywords:** ureteral stricture, metal stents, ureteroplasty, double-J tube, percutaneous nephrostomy

## Abstract

**Background:** The aim of this prospective study was to assess the safety and effectiveness of self-expanding metal ureteral stent (MUS) for the treatment of recurrent ureteral stricture after ureteroplasty.

**Methods:** We prospectively included 24 patients who underwent MUS implantation between February 2019 and August 2020. The inclusion criteria for the procedure were recurrent ureteral strictures after ureteroplasty. A paired *T* test was used to compare continuous variables before and after surgery.

**Results:** A total of 24 patients were finally included in this study. The stricture site was most common on the proximal ureter 19 (79.2%), followed by distal ureter 4 (16.7%) and middle ureter 1 (4.2%). The median length of ureteral stricture is 2.5 (range 1–18) cm. The median operative time was 51.5 min, and the median hospital stay time after surgery was 3 days. Post-operative complication included pain 1 (4.2%), urinary tract infection 2 (8.3%) and hematuria 2 (8.3%). After a median follow-up of 12 months, 19/24 (83.3%) patients were clinically and radiologically successful. We endoscopically adjusted or exchanged the failed stents. The volume of hydronephrosis (124.7 ± 132.5 vs. 66.4 ± 73.2 cm^3^, *P* = 0.015), blood creatinine level (104.5 ± 45.4 vs. 80.1 ± 23.2 μmol/L, *P* = 0.044) and urea nitrogen level (6.9 ± 2.4 vs. 4.8 ± 1.5 mmol/L, *P* = 0.003) decreased significantly after a median follow-up of 12 months.

**Conclusions:** MUS is a safe and effective way to manage recurrent ureteral strictures after ureteroplasty. This technique provides a new choice for the treatment of recurrent stricture.

## Introduction

Ureteral stricture is a complex and challenging disease for urologists. Surgical ureteroplasty including pyeloplasty, ureteroureterostomy, intestinal interposition, oral mucosal graft transplantation, ureteral bladder replantation or autotransplant was selected according to the length and location of strictures (1–3). Although these surgeries are currently the most effective treatments for strictures, recurrent ureteral stricture is still an inevitable trouble (4). Secondary surgical ureteroplasty for recurrent ureteral strictures may be technically difficult and associated with considerable morbidity (5). Thus, a novel procedure is urgent needed to manage recurrent ureteral strictures after ureteroplasty.

Percutaneous nephrostomy tube or double-J tube are widely used to keep drainage for ureteral strictures, but these two methods are accompanied by many complications and need to be replaced regularly (6, 7). Metal ureteral stent (the Resonance stent, the Uventa stent, the Memokath stent and the Allium stent) overcome the above shortcomings and are gradually available for treatment of benign and malignant ureteral strictures. Allium coated Metal ureteral stent (MUS) has far been used in small retrospective series, and it can obtain satisfactory drainage effect (6, 8). However, there is no empirical report about MUS for recurrent ureteral strictures after ureteroplasty. Therefore, we performed an initial single-center experience report about the MUS in management of recurrent ureteral strictures after ureteroplasty.

## Materials and Methods

### Study Population and Data

After receiving approval of Ethics Approval Committee of West China Hospital, we prospectively collect data from patients with recurrent stricture after ureteroplasty who underwent MUS insertion from February 2019 and August 2020. The registration number is 2019-009, and all patients have signed informed consent. Inclusion criteria include patients with recurrent stricture after ureteroplasty (pyeloplasty, ureteroureterostomy, intestinal interposition, oral mucosal graft transplantation, ureteral bladder replantation or autotransplant). The initial causes of ureteral stricture include extrinsic malignant ureteral obstruction, surgical or radiation induced ureteral injury, benign ureteral stricture and retroperitoneal fibrosis. Exclusion criteria include patients with poor basic condition who cannot tolerate surgery; Patients with the transitional cell carcinoma of bladder or ureter; Patients who are unable to maintain the lithotomy position or with severe urethral stricture; Patients with severe urinary tract infection.

We collected the following data: age, sex, body mass index, side, location and length of stricture, surgical history of ureteral stricture, serum creatinine, urea nitrogen levels, hydronephrosis volume, norm GFR of the affected kidney, uptake of affected kidney, operative time, complications, stent number, length of hospital stay, success rate, failure reasons, and symptoms with stents. Abdominal computed tomography (CT) is used to evaluate the volume of hydronephrosis: hydronephrosis volume = length ^*^ width ^*^ depth ^*^ 0.523 (9).

### Surgical Technique

**Figure 1:**
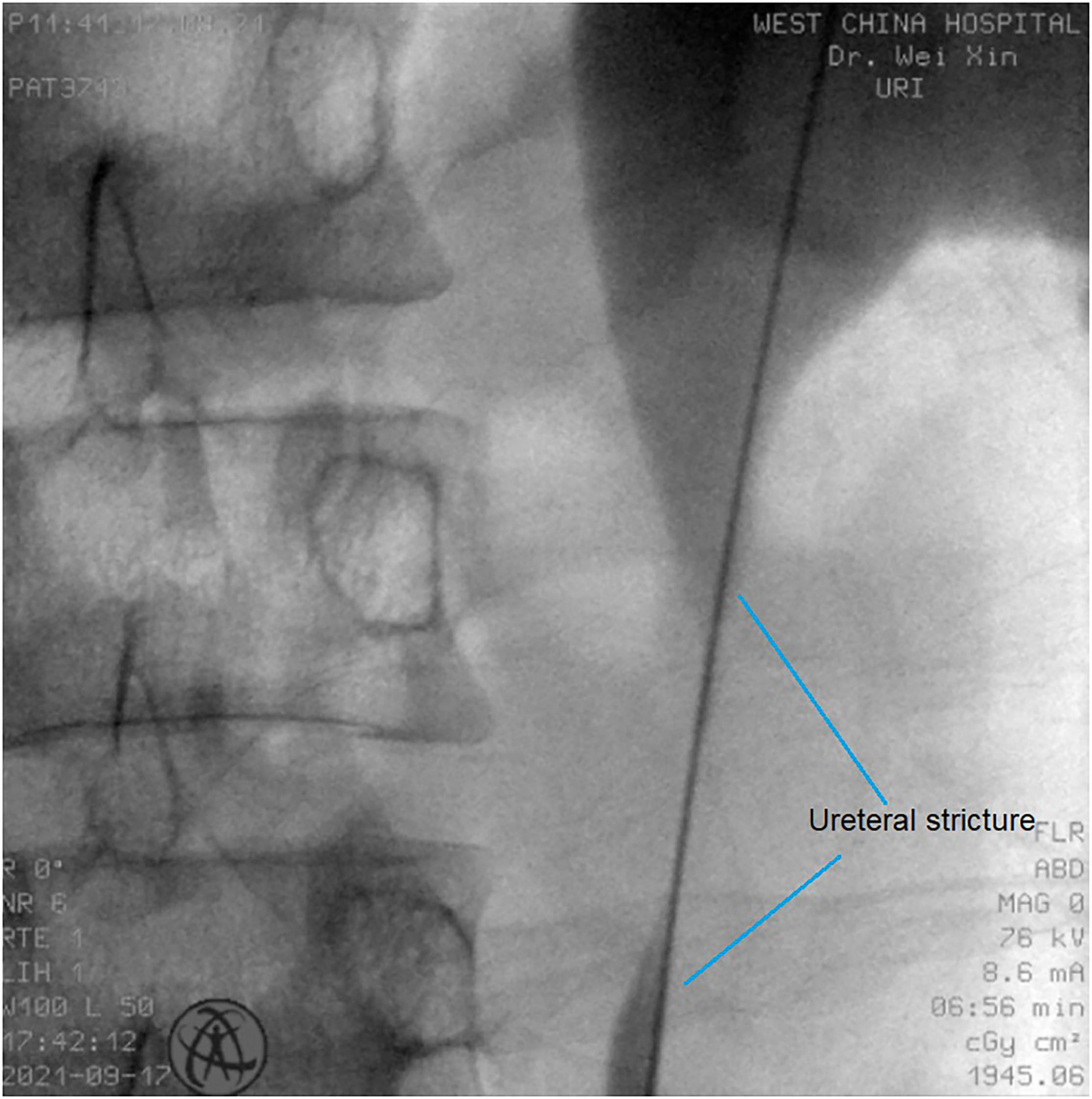
The position and length of ureteral stricture are determined by retrograde or antegrade ureterogram.

**Figure 2:**
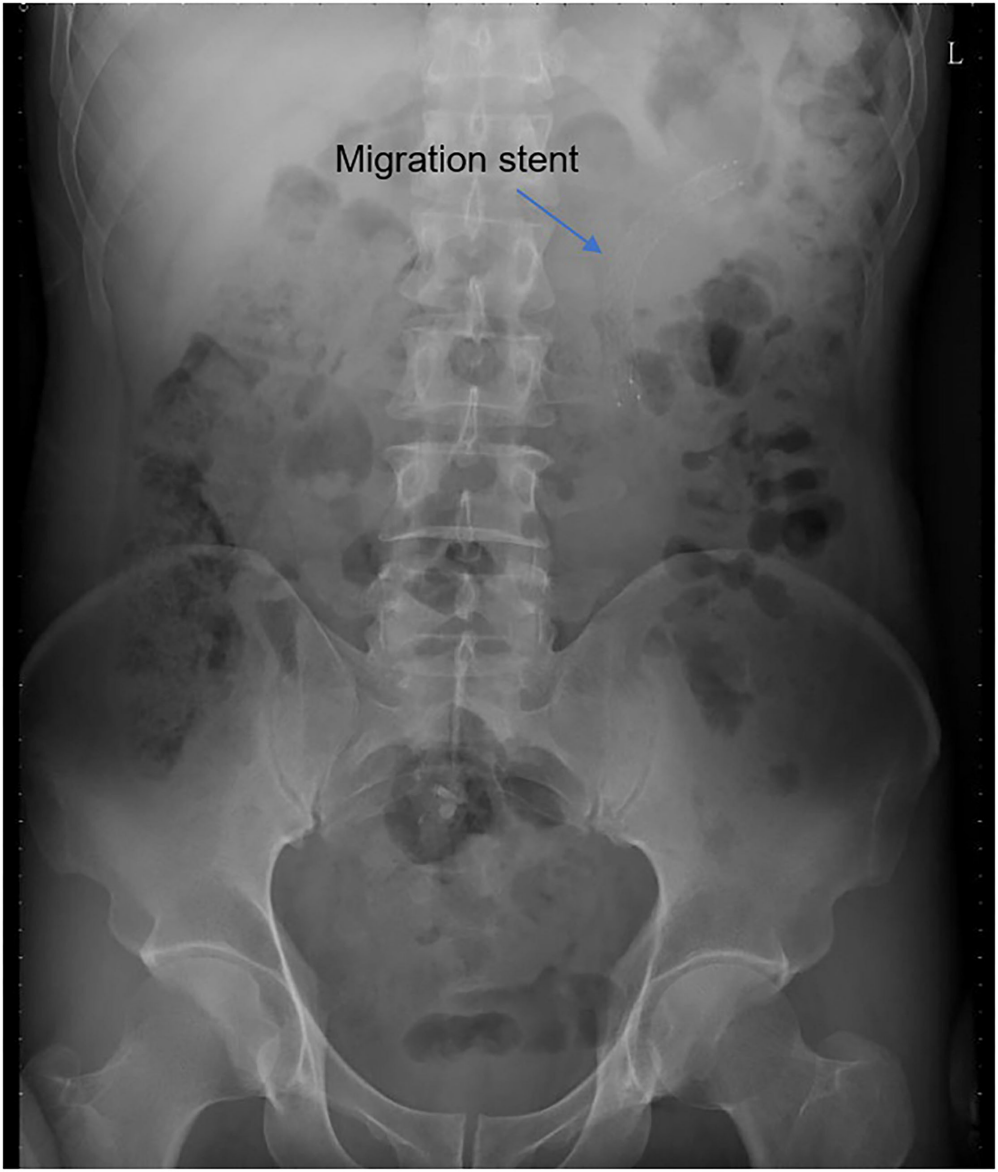
The stent migrated to renal pelvis.

**Figure 3:**
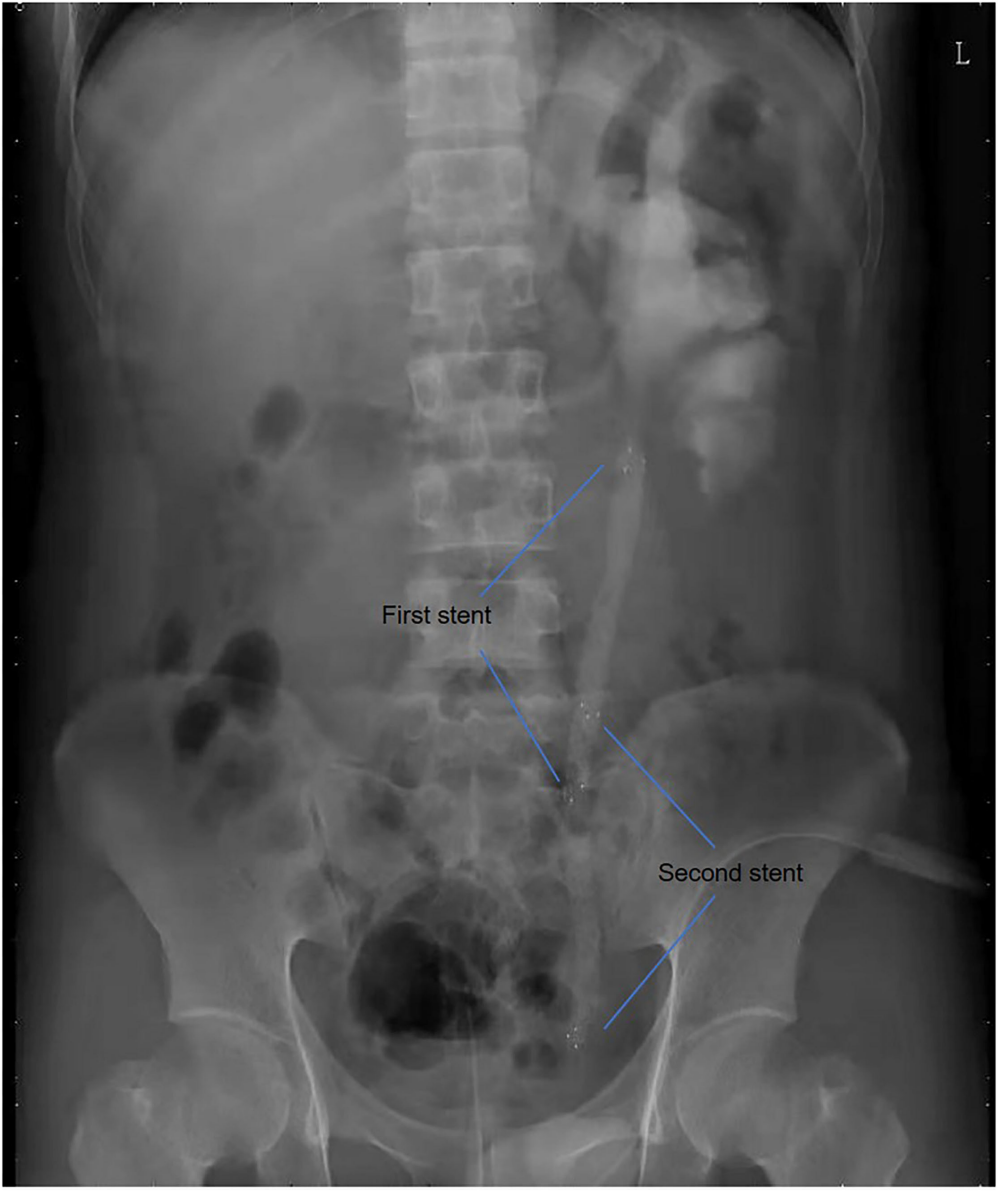
Two stents in tandem for proximal ureteric strictures and long strictures.

All operations were performed by the same endourological skilled surgeon. The surgical procedures were similarly to our previously reported (10, 11). The position and length of ureteral stricture are determined by retrograde or antegrade ureterogram (Figure 1). The two Allium Stents, 10 and 12 cm, are currently available in our hospital. Different stents were selected according to the length of the ureteral strictures. All ureteral strictures were diluted by 18 F or 21 F balloon before the MUS was retrogradely implanted. After confirming that the narrowed segment was dilated satisfactorily, a 24 F or 30 F-coated metal ureteral stent was inserted. We initially implanted one stent for proximal ureteric stricture, and found that the stents migration rate was as high as 80% (Figure 2). So, we improved the surgical technique and inserted two stents in tandem for proximal ureteral strictures and long strictures (Figure 3). When the stent was released satisfactorily, radiography was performed again to confirm the stent position and patency. The migration stents were endoscopically adjusted to the normal position or exchanged as we previous reported (11).

### Follow-Up Protocol

All patients were follow-up every 3 months after surgery, follow-up indicators included blood test, urine test, serum creatinine and urea nitrogen CT of abdomen and single-photon emission computed tomography (SPECT). Record the stent migration time, related treatment procedure and the appearance of various complications. Surgical failure is defined as increased hydronephrosis or deterioration of renal function because of stents migration, occlusion or encrustation.

### Statistical Analysis

The categorical variables were expressed as frequency (proportions), and the continuous variables were described as median (range) or mean ± standard according to skewed distribution or normal distribution, respectively. Statistical comparisons of the continuous data before and after surgery were performed using the paired *T* test, and *p* < 0.05 indicated statistically significant differences. All statistical analysis was performed on SPSS software version 22.0.

## Results

A total of 24 patients with recurrent ureteral stricture after ureteroplasty were performed MUS insertion and included in this study. The general characteristics of patients are listed in Table 1. Among these patients, there were only 4 female patients. The median age of patients was very young, only 37 years old. The stricture site was most common on the proximal ureter 19 (79.2%), followed by distal ureter 4 (16.7%) and middle ureter 1(4.2%). The length of ureteral stricture varies widely by 1–18 cm, and the median length is 2.5 cm. Among surgical history of ureteral recurrent stricture, 14 previously managed with pyeloplasty, 5 repaired by excision of the stricture segment and ureteroureterostomy, 1 followed by ileal ureteral replacement, 2 performed with buccal mucosa graft ureteroplasty and 2 managed with ureteral bladder replantation.

**Table 1:** General characteristics of the patients.

**Variable**	**Overall**
No. patients, *n* (%)	24
Male	21 (87.5)
Female	4 (12.5)
Median age, years (range)	37 (18–62)
BMI, kg/m^2^	23.7 ± 4.2
Side, *n* (%)	
Right	6 (25.0)
Left	18 (75.0)
Median atresia length, cm (range)	2.5 (1–18)
Stricture location, *n* (%)	
Proximal	19 (79.2)
Middle	1 (4.2)
Distal	4 (16.7)
Surgical history of ureteral stricture	
Pyeloplasty	14 (58.3)
Ureteroureterostomy	5 (20.8)
Ileal flap ureteroplasty	1 (4.2)
Buccal mucosa graft ureteroplasty.	2 (8.3)
Ureteral bladder replantation	2 (8.3)

The procedure-related characteristics are shown in Table 2. All recurrent ureteral strictures were successfully inserted MUS stents, and 18 patients inserted two stents in tandem because the ureteral stricture section was too long. The median operative time was 51.5 (range: 20–116) minutes, and the median hospital stay time after surgery was 3 (range: 2–11) days. Post-operative complication included pain 1 (4.2%), urinary tract infection 2 (8.3%) and hematuria 2 (8.3%).

**Table 2:** Procedure-related characteristics.

**Variable**	**Overall**
Success rate, *n* (%)	19/24 (83.3)
Median operative time, min (range)	51.5 (20–116)
Hospital stay time after surgery, day (range)	3 (2–11)
Operative complications, *n*	
Hematuria	1 (4.2)
Pain	2 (8.3)
Urinary tract infection	2 (8.3)
Stent number (tandem), *n* (%)	
1	6 (33.3)
2	18 (66.7)
Median follow-up, month (range)	12 (9–15)
Reasons for failure of surgery, *n* (%)	
Stent migration	3 (12.5)
Stent occlusion	1 (4.2)
Stent encrustation	1 (4.2)
Stent adjusted or exchanged	5 (20.8)
Discomfort with stent	
Gross hematuria after activity	2 (8.3)
Persistent pain	1 (4.2)
Lower urinary tract symptoms	2 (8.3)

After a median follow-up of 12 months, 19/24(83.3%) stents were kept drainage unobstructed. We endoscopically adjusted 3 (12.5%) migration stents to the normal position during follow-up time. Two stents were removed and exchanged because of stents occlusion 1 (4.2%) or encrustation 1 (4.2%). Stent-related complications included gross hematuria after activity 2(8.3%), persistent pain 1(4.2%) and lower urinary tract irritation symptoms 1(4.2%).

Follow up results are listed in Table 3. The volume of hydronephrosis (124.7 ± 132.5 vs. 66.4 ± 73.2 cm^3^, *P* = 0.015), blood creatinine level (104.5 ± 45.4 vs. 80.1 ± 23.2 μmol/L, *P* = 0.044) and urea nitrogen level (6.9 ± 2.4 vs. 4.8 ± 1.5 mmol/L, *P* = 0.003) decreased significantly after a median follow-up of 12 months. However, no significant change was found in the GFR of the affected kidney (25.5 ± 12.6 vs. 25.4 ± 13.6 ml/min/1.73 m^2^, *P* = 0.956) and uptake of the affected kidney (35.1 ± 17.3% vs. 39.2 ± 17.1%, *P* = 0.088) when compared to pre-operation.

**Table 3:** Long-term treatment outcomes of the surgery.

**Variable**	**Pre-operation**	**Last follow-up**	***P***
Hydronephrosis volume/cm^3^	124.7 ± 132.5	66.4 ± 73.2	0.015
Norm GFR of affected kidney (ml/min/1.73 m^2^)	25.5 ± 12.6	25.4 ± 13.6	0.956
Uptake of affected kidney (%)	35.1 ± 17.3%	39.2 ± 17.1%	0.088
Creatinine (μmol/L)	104.5 ± 45.4	80.1 ± 23.2	0.044
Urea nitrogen (mmol /L)	6.9 ± 2.4	4.8 ± 1.5	0.003

## Discussion

As far as we know, this is the first empirical report about MUS for recurrent ureteral strictures after ureteroplasty. After a median follow-up of 12 months, the overall success rate was 83.3%. For surgical failed patients, the stents successfully drainage until the last follow-up after endoscopically adjusted or exchanged of the MUS. In addition, the technique is safe, with a perioperative complication rate of hematuria of 8.3%, urinary tract infection of 8.3% and pain of 4.2%. Moreover, the stent-related complications are fewer and not serious enough to require special treatment.

There are many surgical methods for ureteral strictures, and the specific surgical is selected according to the location and length of the ureteral strictures. Short ureteral strictures <2 cm can be repaired by end-to-end ureteral anastomosis (12). Long distal ureteral strictures (4–5 cm) are ideal for ureteral bladder replantation, while psoas hitch and/or Boari flap are selected for longer strictures up to 6–10 cm or 12–15 cm, respectively (13). Long complex strictures of the proximal or mid ureter are usually managed with bowel or oral mucosal interposition or renal autotransplantation (1). The overall success rate of above-mentioned ureteral reconstruction surgeries exceeds 90% (1, 13). For those patients who have failed ureteral reconstruction surgery, how to deal with recurrent strictures remains a challenge. Buccal mucosal or bowel transplantation for secondary ureteroplasty can achieve good results, but the operation is difficult and there are many complications (4). In this case, it is particularly important to find a management method to relieve the ureteral obstruction and avoid complications.

Minimally invasive management methods for ureteral stricture include percutaneous nephrostomy tube, double-J tube, metal stent and so on (14, 15). Percutaneous nephrostomy tube gets least supported among urologists and patients because of its external nature, reduced quality of life and the most complications (16). The complications of double-J tube are relatively low, but it is very inconvenient because it needs to be replaced every 3 months (17). In these cases, the MUS is the best choice for the recurrent ureteral stricture based on its stronger force for wall support, large-caliber, and long indwelling time. The stents have been fashioned to ensure lumen patency and provide long-term wall support. These stents prevent tissue ingrowth into the lumen by fully covered with a new biocompatible polymer (6). Besides, the MUS is easily endoscopic removal because its special unraveling feature even after a long indwelling period (6).

In this study, the overall success rate at 12 months after surgery is 83.3%, and there are no serious complications related to stents or surgery. The MUS provided the successful clinical use for both benign and malignant ureteral strictures (18–20). Moskovitz et al. reported that their stricture patency rate is 83.7% (40/49) during a mean follow-up period of 21 months, although eight stents are removed endoscopically because of occluded or migration (6). Nevertheless, the patients of the above study are different from ours. We only include cases of recurrent ureteral strictures after ureteroplasty, which are difficult cases to manage. The above results indicate that the success rate of MUS does not reduce in recurrent ureteral strictures. Moreover, for ureteral atresia cases, endoureterotomy by holmium laser was applied to incise the scar tissue, and then the balloon dilation and stent insertion were performed as described before (21).

In our study, 58.3% of patients have suffered failed pyeloplasty. Secondary surgical ureteroplasty for recurrent ureteral strictures may be technically difficult and associated with considerable morbidity. In fact, after pyeloplasty, the renal pelvis usually tortuous and thick. In this case, it is easy to cause stent migration if only one metal stent is implanted. Therefore, we implanted double tandem stents for 18 selected patients.

Previous studies on the MUS reported low rates of complications, including hematuria, pain, urinary tract infections, irritation and encrustation (6, 8, 18–20). In our cohort, pain, urinary tract infection and hematuria are appeared in few patients during the perioperative period. Moreover, these complications are not serious enough and require special medical intervention. Previous studies have reported that proper stent size is particularly important to improve patient comfort (22). In our study, proper stent was selected for each patient to minimize patient discomfort. Thus, the total stent-related complications rate is only 16.7% included gross hematuria after activity, persistent painful and lower urinary tract irritation symptoms, and all the complications arewell-tolerated.

Stent migration is the main complication of metal ureteral stent. Moskovitz and colleagues found that stent migration occurred in 14.2% ureters (1–6 month after insertion) (6). Bahouth et al. reported their experience about 107 stents from 5 different centers, during an average follow-up of 27 months, 10.7% of stents are migration and need to be exchanged (20). Other studies reported that the incidence of stents migration is 18.9% after 7.1 months follow-up time (8). We found that the stents migration incidence in our series is 12.5%, which is similarly to previous reports. The migration stents achieved stricture patency after endoscopically adjusted to the normal position. This indicates that the MUS is effective for recurrent ureteral strictures after ureteroplasty.

Although encouraging, we cannot ignore the limitations when obtaining satisfactory results. One concern is limited by a small cohort that we cannot identify the reason for the failure of the stents. Also, the medium-term follow time does not show the advantages of the MUS for long-term indwelling. Currently, we are working on a large-scale prospective study to assist with addressing these issues, and further assess the long-term safety and effectiveness of the MUS.

## Conclusions

MUS is a safe and effective way to manage recurrent ureteral strictures after ureteroplasty. This technique provides a new choice for the treatment of recurrent stricture.

## Data Availability Statement

The original contributions presented in the study are included in the article/supplementary material, further inquiries can be directed to the corresponding author/s.

## Ethics Statement

The studies involving human participants were reviewed and approved by Ethics Approval Committee of West China Hospital, and the registered number is 2019-009. The patients/participants provided their written informed consent to participate in this study. Written informed consent was obtained from the individual(s) for the publication of any potentially identifiable images or data included in this article.

## Author Contributions

XG wrote the manuscript writing. JC, ZJ, and WW collected the data. LP, MW, and ZL analyzed the data. XW helped design the study and revise article. All authors have read and approved the manuscript.

## Funding

This study was supported by the New Clinical Technology in West China Hospital of Sichuan University (Grant No. 20HXJS002).

## Conflict of Interest

The authors declare that the research was conducted in the absence of any commercial or financial relationships that could be construed as a potential conflict of interest.

## Publisher's Note

All claims expressed in this article are solely those of the authors and do not necessarily represent those of their affiliated organizations, or those of the publisher, the editors and the reviewers. Any product that may be evaluated in this article, or claim that may be made by its manufacturer, is not guaranteed or endorsed by the publisher.
